# An oral toxicity test in rats and a genotoxicity study of extracts from the stems of *Opuntia ficus-indica* var. *saboten*

**DOI:** 10.1186/s12906-019-2442-7

**Published:** 2019-01-28

**Authors:** Eun Hye Han, Mi Kyung Lim, Sang Ho Lee, Md. Mahbubur Rahman, Young-Hee Lim

**Affiliations:** 1 Department of Integrated Biomedical and Life Sciences, Graduate School, Korea University, Seoul, 02841 Republic of Korea; 2 Research and Development Center, Koreaeundan, Seongnam, Gyeonggi-Do 13207 Republic of Korea; 3 KNOTUS Co., Ltd. Research Center, 189 Donggureung-Ro, Guri-Si, Gyeonggi-Do 11906 Republic of Korea; 4 Department of Public Health Science (Brain Korea 21 PLUS program), Graduate School, Korea University, Seoul, 02841 Republic of Korea; 5 Department of Laboratory Medicine, Korea University Guro Hospital, Seoul, 08308 Republic of Korea

**Keywords:** *Opuntia ficus-indica* var. *saboten*, Mutagenicity, Ames test, Chromosomal aberration assay, Micronucleus, Genotoxicity

## Abstract

**Background:**

*Opuntia ficus-indica* var. *saboten* (OFIS) is used widely in Korea to treat constipation due to its diuretic effects and its enhancement of bowel function and appetite. However, its safety has not yet been established. The aim of this study was to evaluate the repeated oral toxicity and genotoxicity of OFIS extract (OE).

**Methods:**

White female and male Sprague Dawley rats (*n* = 6) were divided into 4 groups, and OE was administered to them orally (0, 500, 1000, and 2000 mg/kg/day, respectively) for one week. The Ames test, the chromosomal aberration assay, and the mammalian micronucleus test were performed to determine the OE genotoxicity. The Ames test was conducted using *Salmonella typhimurium* (*S. typhimurium*) strains TA100, TA1535, TA98, and TA153 and *Escherichia coli* (*E. coli*) WP2 urvA, and Chinese hamster lung (CHL) cells were used for the chromosomal aberration assay. The mammalian micronucleus test was performed using mouse bone marrow cells.

**Results:**

This study revealed that OE administration did not alter the normal rat behavior, body weight gain, and food and water consumption with respect to the normal controls. In addition, there were no toxic effects observed during the ophthalmological test. The biochemical hematological and serum values as well as urinalysis parameters and organ weights were all similar to those of the normal control group. In addition, no mutagenicity effects from the OE were found in *S. typhimurium* or *E. coli* with or without S9 activation according to the Ames test. The OE did not significantly alter the number of structural aberrations in the CHL cells in the presence or absence of S9 activation. The oral administration of OE also caused no significant increase in the number of micronucleated polychromatic erythrocytes or in the mean ratio of polychromatic to total erythrocytes.

**Conclusions:**

In conclusion, OE could be considered as a reliable and safe herbal medicine or functional food since no toxicity was found under the conditions of this study.

## Background

*Opuntia ficus-indica* var. *saboten* (OFIS) is a tropical perennial cactus belonging to the Cactaceae family and Opuntia genus [1]. Many of the biologically active phytochemicals in OFIS have been identified, including dihydrokaempferol, dihydroquercetin, kaempferol 3-O-rutinoside, kaempferol 7-O-glucoside, quercetin, quercetin 3-O-galactoside, 3-O-methylquercetin, isorhamnetin, isorhamnetin 3-O-glucoside, isorhamnetin 3-O-galactoside, and narcissin [2, 3]. The various parts of OFIS including the fruits, flowers, stem, skin, and cladode have been studied for their health benefits due to their abundance of vitamins, minerals, antioxidants, and polyphenols. Several hypoglycemic, hypolipidemic anti-ulcerogenic, anti-ulcer, antioxidative, anti-cancer, neuroprotective, hepatoprotective, and anti-proliferative properties of OFIS have previously been reported [4, 5]. OFIS has also been used for a long time in Korea as a constipation treatment due to its diuretic effects and enhancement of bowel function and appetite. Stems have been used as folk remedies for skin diseases, rheumatism, and burns [5, 6]. In addition to these effects, OFIS extract (OE) can reportedly improve memory [7]. OFIS is cultivated in the southern part of Korea. It grows well in any soil, and mass production is possible [8]. However, unlike a variety of OFIS extracts, refined foods, and processed foods sold around the world, only processed foods such as chocolate and tea are produced mainly in the Jeju area.

Toxicity testing provides an important part of the basis for public health and regulatory decisions concerning the safety of herbal medicines. Toxicity test methods were developed incrementally using laboratory animals (e.g., rats, mice, and rabbits) and cultured cells in vitro [9]. In addition, genotoxicity evaluation is an excellent test to describe a risk assessment of chemical substances for human health and is necessary to identify their clastogenic and mutagenic potentials [10]. Genotoxicity tests describe chemical or medicinal agents according to their potential to damage genetic information within a cell by causing mutations, which may lead to cancer. The purpose of this study is to confirm the safety of herbal products [10–12]. Although the phytochemicals in OFIS have been extensively studied in Korea [2, 3] and the extract has a long history of use, its natural origin and widespread use do not ensure its safety. To the best of our knowledge, there have been no previous reports of toxicity tests on OFIS. Therefore, the aim of this study is to evaluate the general toxicity and genotoxicity of OFIS extracts (OE) using several tests including the Ames test, a chromosomal aberration test, and a mammalian micronucleus test to better understand the safety of OFIS cultivated on Jeju Island.

## Methods

### Preparation of OFIS stem extract

The OFIS stem was cultivated and collected from Jeju Island, Korea in April (spring) by SK Bioland in Gyeonggi, Korea. The plant material used in this study was identified by Dr. Yu HJ from SK Bioland and ultimately confirmed by Prof. Lim YH. The OFIS extract was prepared as described previously [2]. In brief, powder obtained by pulverizing 5 kg of the dried product and 75 L of 70% alcohol were placed in a 200 L fermenter and extracted with stirring at 80 °C for 2 h. The extract was filtered, transferred to a concentrator, and concentrated. Then, an equal amount of dextrin was added and the product was dried with a spray drier until a final product (extract) was prepared. This extract was freshly dissolved in sterile water for injection to make different concentrations for oral administration. The plant material was deposited in Koreaeundan Co., Ltd. The voucher specimen/deposition number is OFP-D006.

### Experimental animals

Sprague-Dawley rats were obtained from Sam Taco Bio Korea (Osan, Korea) to be used in this experiment. The animals were housed in a feeding room that was maintained at a specific temperature (23 ± 3 °C), relative humidity (55 ± 15%), ventilation frequency (10–20 times/h), and lighting (12 h; off from 8:00 a.m. to 8:00 p.m.). Food and water were available ad libitum. After a 7-day acclimatization period, OE was administered orally to the female and male rats, and they were randomly distributed into four groups (*n* = 6) according to the dose (0, 500, 1000, and 2000 mg/kg/day). Each rat received a daily dose for one week to evaluate the potential toxicity. All the experimental protocols employed herein were approved by the committee on the care of laboratory animal resources at Chemon Co, Ltd. (registration number: 16-RR-563 N).

### Observation of general symptoms: weight, feed, and water intake

All the rats were closely observed to identify any indication of toxicity within the treatment period and ultimately before sacrifice at the 8th day. Observations of rat mortality, weakness, aggressiveness, feed and water consumption, diarrhea or loose feces, salivation, noisy breathing, and clonic convulsion were recorded once daily. The body weight for each rat was measured two times, before OE administration and before sacrifice. The appearance of their eyes was visually observed (for any discharge from the eyes, for changes in the normal color) in all the animals during the observation period.

### Sample collection

Urinalysis was performed by collecting approximately 0.3 mL of fresh urine from one animal in a sterilized stainless-steel breeding box. All the animals were fasted overnight on the last day, the 7th day, and on the morning of the 8th day, the rats were sacrificed using anesthesia in an induction chamber with isoflurane 3.5% for 2–5 min. Blood was collected from the posterior vena cava using a syringe, and hematological and blood biochemical tests were performed to analyze the hematological parameters. After the blood was collected, the abdominal aorta and the posterior vena cava were cut to check for hemorrhagic cadavers. All of the organs of the body surface as well as the subcutaneous, thoracic, and abdominal cavities were observed, and the autopsy findings were recorded. Finally, the organ weights were measured and preserved.

### Genotoxicity test

#### Bacterial reverse mutation test (Ames test)

To confirm the mutagenicity of OE, the histidine operon-containing strains, *S. typhimurium* strains TA100, TA1535, TA98, and TA1537 [13] and tryptophan strain *E. coli* WP2 urvA [14] were used for the experiments. The strains were obtained from Molecular Toxicology Inc. (Boone, NC 28607, USA). The OE was prepared by suspending a 70% extract of OFIS stems in sterilized water and then autoclaving it at 121 °C for 20 min. Just prior to treatment, the suspension was diluted with the same container and used in the post-fabrication test by concentration. The test groups consisted of 5, 15, 50, 150, 500, 1500, 3000, and 5000 μg/plate of OE and were treated with an S9 mixture (the rat livers), including negative and positive controls. Each group was divided into S9 mixture-untreated groups and S9 mixture-tested groups using two plates. The positive control substances were 2-aminoanthracene (2-AA), benzo [a] pyrene (B [a] P) and 2-nitrofluorene (2-NF), 4-nitroquinoline-1-oxide (4NQO), and acridine mutagen ICR 191 (ICR-191). After the test materials and strain were dispensed into the medium, the cells were incubated at 37 ± 2 °C for 50 ± 2 h, and the mutation-containing colonies were counted visually. The fine colony formation of the basal growth layer was observed and compared with the negative control group. When the test material was mixed with test agar and top agar, precipitate formation was also observed. Cytotoxicity can be identified for confirmation when the basal growth layer becomes thinner and the number of colonies decreases.

#### Chromosome aberration testing using Chinese hamster lung (CHL) cells

The experiment was performed using a CHL/IU cell line derived from female hamster lung fibroblasts. The CHL cells were purchased from the American Type Culture Collection (Manassas, VA, USA), cultured in minimal essential medium (MEM), and supplemented with 10% fetal bovine serum (FBS, HyClone, USA), GlutaMax-1 supplement, and penicillin-streptomycin in a 37 ± 1 °C incubator at 5% CO_2_. Subcultures were created every 2 days using 0.1% trypsin-EDTA solutions. The OE was freshly prepared as before. The treatment methods were classified into three types according to the application and nonuse of the metabolic active system. Treatment 1 was performed for 6 h using a metabolic activation system, and 18 h of recovery time were allowed to observe the chromosomal aberrations. Treatments 2 and 3 were performed for 6 h, followed by an 18-h recovery, 24-h treatment, and 0-h recovery without the use of the metabolic activation system. In Test 1, the test substances were applied at concentrations of 500, 1500, 3000, 3500, 4000, 4500, and 5000 μg/mL, including the negative control. Treatment 2 was applied at 500, 1500, 1800, 2000, and 3000 μg/mL along with a negative control, and treatment 3 involved 500, 1500, 3000, 4000, and 5000 μg/mL concentrations and a negative control. At 24 h after the start of the test substance treatment, 50 μL of colchicine solution was used to collect medium-sized cells. After the media along with the mid-term cells were centrifuged at 200×*g* for 5 min, the cells were stored (in 75 mM KCl solution) and fixed (methyl alcohol: glacial acetic acid = 3: 1 *v*/v). Chromosomal anomalies were counted at 1000-fold magnification by staining with 5% Giemsa solution. The frequency of chromosomal induction was expressed as the percentage of mid-term cells with chromosomal abnormalities.

#### Micronucleus test using mouse bone marrow cells

Twenty-four male ICR mice were used in the experiment and were raised under the same conditions as the single dose toxicity test. Cyclophosphamide monohydrate (CPA) was used as a positive control, and four animals were assigned to each group, including the negative control group and the positive control group. The OE was administered orally for 2 days by dissolving OE in the sterilized water to make final concentrations of 500, 1000, 2000, and 5000 mg/kg/day. The positive control substance (70 mg/kg) was dissolved in the physiological saline solution immediately before compound administration and was administered intraperitoneally once after preparation. The highest dose was set at 5000 mg/kg considering that 70% of the extract from the OFIS stem was used as a raw material in a functional health food. The general symptoms were recorded by individual observation, and the body weights were measured before, during, and at the time of preparation for all the animals. The method described in Hayashi (1983) [15] was applied to confirm the formation of micronuclei. The bone marrow cells were fixed with methanol according to the method described in Schimid (1975) [16] and stained with red fluorescent stain using acridine orange, a fluorescent stain solution. The polychromatic erythrocytes (PCE) with micronuclei were counted while counting out 2000 polychromatic erythrocytes per animal under a fluorescence microscope. To determine the cytotoxicity regardless of the presence or absence of micronuclei, the PCE: red blood cell (RBC) ratio was calculated by counting the red cells (RBCs, polyglutathione erythrocytes).

### Statistical analyses

Bonferroni’s post-hoc test was used following a one-way ANOVA for the weight, hematology, blood biochemical tests, and long-term weights, and the homogeneity of variance was tested using Levene’s test. The urine test results were analyzed using a nonparametric Kruskal-Wallis test. The nonparametric Kruskal-Wallis test was performed on the micronucleus frequency using SPSS Statistics 22 for Medical Science in the micronucleus test, and the negative control and positive control data were analyzed using a Mann-Whitney U test. A one-way ANOVA was applied to the RBC ratio and body weight, and the homogeneity of variance was tested using Levene’s test. The mean difference between the negative and positive controls was analyzed using the Student’s t-test.

## Results

### General symptoms, weight, feed and water intake and clinical pathology

There were no changes in the general symptoms, body weight changes, water intake, feed intake, urine test or ophthalmological test in the rats treated with the OE compared with the normal control group (Tables 1 and 2). In the hematologic test, the red blood cell distribution width (RDW) was significantly higher in females treated at 2000 mg/kg/day (*P* < 0.05) when compared with the vehicle-treated female rat group, but it was estimated to be within the range of historical reference data (Table 3). While the blood biochemical tests showed that the triglyceride (TG) levels were significantly lower (*P* < 0.05) in females treated at 1000 mg/kg/day, dose-response correlations were not observed. In addition, the total bilirubin (TBIL) and alanine amino transferase (ALT) levels were significantly higher in females treated with 2000 mg/kg/day (*P* < 0.01) (Table 4). This finding was presumed to be due to the test substance, but the liver weight and autopsy findings were not different from those of the vehicle control group. The blood urea nitrogen (BUN) values were significantly higher in males (*P* < 0.01) treated with OE when compared to vehicle-treated male rats, but dose-response correlations were not observed (Table 4). The autopsy findings showed no change in long-term weight due to the OE, and no gross abnormal findings were observed.

**Table 1:** Effect of oral administration of OE on body weight, food and water consumption, and relative organs weight to body weight

	Male				Female			
	G1	G2	G3	G4	G1	G2	G3	G4
IBW (g)	200.81 ± 12.55	198.09 ± 7.90	201.98 ± 8.35	202.77 ± 12.53	144.92 ± 5.29	149.25 ± 6.00	153.67 ± 9.68	160.69 ± 6.01
FBW (g)	220.79 ± 15.32	211.72 ± 7.37	219.15 ± 10.37	217.26 ± 9.93	161.83 ± 2.88	166.26 ± 7.00	173.32 ± 12.22	173.29 ± 3.79
Food consumption g/day	20.91 ± 1.11	19.46 ± 1.20	20.14 ± 0.91	20.11 ± 0.79	12.95 ± 1.32	14.61 ± 1.01	15.39 ± 0.31	15.10 ± 0.05
Water consumption mL/day	22.83 ± 1.13	24.14 ± 0.82	24.42 ± 0.35	25.27 ± 0.08	22.10 ± 0.93	17.26 ± 1.42	20.95 ± 0.35	22.03 ± 0.73
Adrenal gland-left	0.0193 ± 0.003	0.0174 ± 0.001	0.0214 ± 0.002	0.0195 ± 0.004	0.0212 ± 0.003	0.0224 ± 0.002	0.0247 ± 0.004	0.0217 ± 0.002
% to BW	0.009 ± 0.001	0.008 ± 0.001	0.010 ± 0.001	0.009 ± 0.002	0.015 ± 0.001	0.015 ± 0.001	0.016 ± 0.002	0.014 ± 0.001
Adrenal gland-right	0.017 ± 0.002	0.018 ± 0.001	0.021 ± 0.003	0.019 ± 0.003	0.021 ± 0.004	0.021 ± 0.002	0.024 ± 0.005	0.022 ± 0.002
% to BW	0.008 ± 0.001	0.008 ± 0.000	0.009 ± 0.001	0.009 ± 0.002	0.015 ± 0.002	0.014 ± 0.001	0.015 ± 0.003	0.014 ± 0.001
Thymus	0.631 ± 0.076	0.572 ± 0.168	0.694 ± 0.105	0.614 ± 0.057	0.368 ± 0.046	0.438 ± 0.043	0.385 ± 0.041	0.441 ± 0.052
% to BW	0.285 ± 0.016	0.271 ± 0.080	0.316 ± 0.040	0.283 ± 0.023	0.255 ± 0.042	0.293 ± 0.019	0.250 ± 0.015	0.276 ± 0.043
Spleen	0.697 ± 0.058	0.574 ± 0.091	0.653 ± 0.045	0.616 ± 0.028	0.434 ± 0.078	0.411 ± 0.023	0.423 ± 0.072	0.467 ± 0.032
% to BW	0.316 ± 0.007	0.272 ± 0.050	0.298 ± 0.008	0.284 ± 0.024	0.299 ± 0.046	0.276 ± 0.021	0.275 ± 0.041	0.290 ± 0.009
Kidney-left	0.877 ± 0.079	0.797 ± 0.044	0.851 ± 0.034	0.895 ± 0.011	0.624 ± 0.085	0.596 ± 0.025	0.619 ± 0.058	0.653 ± 0.083
% to BW	0.397 ± 0.008	0.377 ± 0.031	0.388 ± 0.007	0.412 ± 0.014	0.430 ± 0.051	0.399 ± 0.017	0.403 ± 0.026	0.406 ± 0.043
Kidney-right	0.889 ± 0.056	0.818 ± 0.064	0.876 ± 0.012	0.897 ± 0.010	0.637 ± 0.069	0.588 ± 0.008	0.608 ± 0.062	0.667 ± 0.057
% to BW	0.403 ± 0.010	0.387 ± 0.037	0.400 ± 0.017	0.414 ± 0.022	0.440 ± 0.044	0.395 ± 0.021	0.396 ± 0.029	0.415 ± 0.026
Heart	0.945 ± 0.161	0.782 ± 0.042	0.868 ± 0.047	0.905 ± 0.069	0.620 ± 0.015	0.620 ± 0.024	0.626 ± 0.066	0.620 ± 0.068
% to BW	0.426 ± 0.046	0.370 ± 0.033	0.396 ± 0.008	0.416 ± 0.020	0.428 ± 0.023	0.415 ± 0.002	0.409 ± 0.048	0.386 ± 0.038
Lung	1.243 ± 0.111	1.048 ± 0.081	1.138 ± 0.079	1.159 ± 0.082	0.912 ± 0.042	0.882 ± 0.104	0.950 ± 0.105	1.031 ± 0.110
% to BW	0.562 ± 0.012	0.495 ± 0.043	0.519 ± 0.034	0.534 ± 0.043	0.629 ± 0.018	0.590 ± 0.052	0.620 ± 0.076	0.641 ± 0.058
Brain	1.918 ± 0.027	1.825 ± 0.049	1.858 ± 0.108	1.904 ± 0.027	1.741 ± 0.044	1.825 ± 0.084	1.788 ± 0.065	1.750 ± 0.113
% to BW	0.871 ± 0.049	0.863 ± 0.047	0.849 ± 0.047	0.877 ± 0.029	1.202 ± 0.043	1.223 ± 0.008	1.168 ± 0.111	1.090 ± 0.083
Liver	7.564 ± 1.085	6.768 ± 0.356	7.725 ± 0.576	7.464 ± 0.520	4.915 ± 0.143	4.815 ± 0.518	4.830 ± 0.213	5.158 ± 0.200
% to BW	3.417 ± 0.259	3.196 ± 0.097	3.531 ± 0.334	3.439 ± 0.257	3.393 ± 0.073	3.223 ± 0.253	3.147 ± 0.119	3.211 ± 0.082

G1: negative control; G2: 500 mg/kg OE treated group; G3: 1000 mg/kg OE treated group; G4: 2000 mg/kg OE treated group. IBW: initial body weight; FBW: final body weight

Data are expressed as mean ± SD. Bonferroni’s post hoc test was used following a one-way ANOVA vs. G1, and the homogeneity of variance was tested using Levene’s test. There was no significance difference among all the groups

**Table 2:** Effect of oral administration of OE on urinalysis parameters in male and female rats

Tests	Result	Grade	Male				Female			
			G1	G2	G3	G4	G1	G2	G3	G4
GLU	–	0	3	3	3	3	3	3	3	3
BIL	–	0	3	3	3	3	3	3	3	3
KET	–	0	2	2	2	0	3	3	3	2
	+/−	1	1	1	1	3	0	0	0	1
SG	≤1.005	0	0	0	0	0	1	0	0	0
	1.010	1	0	0	1	0	2	1	1	0
	1.015	2	0	0	0	1	0	1	2	1
	1.020	3	0	2	1	0	0	1	0	1
	1.025	4	3	1	1	1	0	0	0	1
	≥1.030	5	0	0	0	1	0	0	0	0
pH	6.5	0	1	0	0	1	0	0	0	0
	7.0	1	0	2	0	1	0	0	0	0
	7.5	2	1	0	1	0	3	3	3	2
	8.0	3	1	0	2	1	0	0	0	1
	8.5	4	0	1	0	0	3	3	3	1
PRO	–	0	0	0	1	0	0	0	0	2
	+/−	1	0	0	1	1	3	3	3	3
	1+	2	3	3	1	2	3	3	3	3
URO	0.2	0	3	3	3	3	3	2	2	3
NIT	–	0	3	3	3	3	0	0	1	0
BLO	–	0	3	3	3	3	0	1	0	0
LEU	–	0	2	1	1	0	3	3	3	3
	+/−	1	0	2	1	1	3	3	3	3
	1+	2	1	0	1	2	3	3	3	3
Clarity	–	0	3	3	3	3	3	3	3	3
Color-Yellow			3	3	3	3	3	3	3	3

G1: negative control; G2: 500 mg/kg OE treated group; G3: 1000 mg/kg OE treated group; G4: 2000 mg/kg OE treated group. GLU: glucose (mg/dL); BIL: bilirubin (mg/dL); KET: ketone bodies (mg/dL); SG: specific gravity; PRO: protein (mg/dL); URO: urobilinogen (mg/dL); NIT: nitrites (mg/dL); BLO: Occult blood: LEU: leukocytes (Leu/dL). Results were analyzed using the non-parametric Kruskal-Wallis test but there were no significance differences among the groups

**Table 3:** Effect of oral administration of OE on hematological parameters

Tests	Male				Female			
	G1	G2	G3	G4	G1	G2	G3	G4
RBC (10^6^/μL)	6.91 ± 0.43	7.23 ± 0.27	6.85 ± 0.38	6.81 ± 0.17	7.05 ± 0.16	7.25 ± 0.25	7.05 ± 0.08	6.89 ± 0.31
HGB (g/dL)	13.5 ± 0.6	14.2 ± 0.2	13.6 ± 0.7	13.7 ± 0.3	13.9 ± 0.2	14.1 ± 0.3	13.8 ± 0.1	13.6 ± 0.7
HCT (%)	40.8 ± 2.0	42.7 ± 0.8	41.3 ± 1.9	40.5 ± 0.6	40.7 ± 0.3	41.7 ± 0.7	40.6 ± 0.3	40.1 ± 1.6
MCV (fL)	59.1 ± 0.9	59.1 ± 1.1	60.3 ± 0.8	59.5 ± 0.6	57.8 ± 1.0	57.6 ± 1.0	57.5 ± 0.8	58.2 ± 0.5
MCH (pg)	19.5 ± 0.3	19.6 ± 0.5	19.8 ± 0.5	20.1 ± 0.5	19.8 ± 0.4	19.4 ± 0.4	19.6 ± 0.3	19.7 ± 0.2
MCHC (g/dL)	32.9 ± 0.2	33.2 ± 0.2	32.9 ± 1.0	33.8 ± 0.8	34.3 ± 0.5	33.7 ± 0.4	34.0 ± 0.2	33.9 ± 0.7
RDW (%)	12.6 ± 0.8	11.8 ± 0.1	12.9 ± 0.7	12.3 ± 0.3	11.4 ± 0.3	11.4 ± 0.3	11.4 ± 0.4	12.2 ± 0.3*
HDW (g/dL)	2.32 ± 0.07	2.21 ± 0.05	2.22 ± 0.04	2.22 ± 0.07	2.16 ± 0.08	2.17 ± 0.10	2.09 ± 0.06	2.24 ± 0.05
PLT (10^3^/μL)	1404.7 ± 280.7	1196.7 ± 292.9	1274.3 ± 31.8	1149.7 ± 133.6	1078.0 ± 128.6	1091.7 ± 72.0	1132.3 ± 80.1	1204.7 ± 88.4
MPV (fL)	5.50 ± 0.17	5.57 ± 0.23	5.53 ± 0.23	5.60 ± 0.17	5.40 ± 0.17	5.67 ± 0.21	5.40 ± 0.26	5.37 ± 0.06
WBC (10^3^/μL)	5.56 ± 2.15	6.23 ± 1.90	7.40 ± 3.07	5.63 ± 1.12	4.33 ± 1.75	4.89 ± 1.32	3.75 ± 0.78	4.86 ± 1.65
NEU (10^3^/μL)	0.4 ± 0.1	0.5 ± 0.1	0.5 ± 0.2	0.4 ± 0.2	0.3 ± 0.1	0.5 ± 0.3	0.3 ± 0.1	0.4 ± 0.2
LYM (10^3^/μL)	5.0 ± 1.9	5.5 ± 1.7	6.6 ± 2.7	5.0 ± 0.9	3.8 ± 1.6	4.1 ± 1.3	3.3 ± 0.7	4.3 ± 1.3
MON (10^3^/μL)	0.15 ± 0.09	0.14 ± 0.05	0.19 ± 0.10	0.16 ± 0.04	0.10 ± 0.05	0.10 ± 0.06	0.08 ± 0.04	0.09 ± 0.03
EOS (10^3^/μL)	0.03 ± 0.01	0.04 ± 0.02	0.04 ± 0.01	0.03 ± 0.01	0.06 ± 0.02	0.08 ± 0.05	0.05 ± 0.01	0.08 ± 0.04
BAS (10^3^/μL)	0.01 ± 0.01	0.01 ± 0.01	0.02 ± 0.01	0.01 ± 0.01	0.00 ± 0.01	0.00 ± 0.00	0.01 ± 0.01	0.01 ± 0.01
LUC (10^3^/μL)	0.04 ± 0.02	0.03 ± 0.01	0.06 ± 0.04	0.03 ± 0.01	0.02 ± 0.02	0.03 ± 0.01	0.03 ± 0.02	0.03 ± 0.01

G1: negative control vehicle treated group; G2: 500 mg/kg OE treated group; G3: 1000 mg/kg OE treated group; G4: 2000 mg/kg OE treated group

RBC: erythrocyte; HGB: hemoglobin: HCT: hematocrit; MCV: mean corpuscular volume: MCH: mean corpuscular hemoglobin; MCHC: mean corpuscular hemoglobin concentration; RDW: Red cell distribution width; HDW: Hemoglobin distribution width; PT: Platelet; MPV: Mean platelet volume; WBC: white blood cell; NEU: Neutrophil; LYM: Lymphocyte; MON: Monocyte; EOS: Eosinophil; BAS: Basophils; LUC: Large unstained cell. Data are expressed as mean ± SD. *Significant difference at *P* < 0.05 levels compared with the G1 group. Bonferroni’s post hoc test was used following a one-way ANOVA vs. G1, and the homogeneity of variance was tested using Levene’s test

**Table 4:** Effect of oral administration of OE on serum biochemical profiles

Tests	Male				Female			
	G1	G2	G3	G4	G1	G2	G3	G4
AST (U/L)	118.1 ± 15.9	110.0 ± 15.5	102.6 ± 13.2	114.3 ± 10.4	80.5 ± 11.0	81.3 ± 3.4	84.3 ± 14.7	85.9 ± 11.4
ALT (U/L)	27.0 ± 3.9	32.9 ± 7.5	27.4 ± 4.4	29.5 ± 4.2	21.6 ± 2.6	22.3 ± 3.2	18.0 ± 1.0	34.1 ± 2.4**
ALP (U/L)	190.4 ± 5.3	213.1 ± 39.1	192.3 ± 3.5	210.2 ± 42.6	135.0 ± 41.5	121.7 ± 13.3	126.8 ± 23.6	145.6 ± 27.3
CPK (U/L)	763.0 ± 156.9	578.3 ± 113.2	525.0 ± 16.6	628.3 ± 227.0	216.0 ± 14.7	232.0 ± 63.0	262.3 ± 217.2	181.0 ± 68.1
TBIL (mg/dL)	0.11 ± 0.02	0.11 ± 0.01	0.12 ± 0.02	0.11 ± 0.02	0.11 ± 0.01	0.11 ± 0.02	0.10 ± 0.02	0.14 ± 0.01*
GLU (mg/dL)	97.6 ± 5.4	110.7 ± 13.2	122.4 ± 27.7	108.1 ± 29.4	117.1 ± 6.4	122.0 ± 5.2	120.2 ± 3.5	110.2 ± 11.4
TCHO (mg/dL)	84.0 ± 10.4	75.7 ± 3.2	82.7 ± 3.8	79.3 ± 13.7	72.7 ± 9.0	75.7 ± 7.6	72.7 ± 8.3	77.3 ± 10.1
TG (mg/dL)	99.3 ± 7.6	74.7 ± 13.8	95.3 ± 25.7	88.3 ± 17.2	37.3 ± 5.0	28.0 ± 4.0	25.7 ± 5.0*	42.7 ± 6.0
TP (g/dL)	5.44 ± 0.19	5.40 ± 0.26	5.50 ± 0.14	5.44 ± 0.29	5.53 ± 0.10	5.42 ± 0.19	5.49 ± 0.25	5.33 ± 0.19
Albumin (g/dL)	2.92 ± 0.10	2.89 ± 0.09	2.93 ± 0.09	2.89 ± 0.13	3.05 ± 0.05	3.02 ± 0.12	3.04 ± 0.11	2.97 ± 0.14
A/G ratio	1.16 ± 0.02	1.16 ± 0.05	1.14 ± 0.06	1.14 ± 0.04	1.23 ± 0.05	1.26 ± 0.03	1.25 ± 0.06	1.26 ± 0.07
BUN (mg/dL)	9.6 ± 0.9	13.2 ± 0.8**	14.9 ± 1.5**	13.0 ± 1.4**	15.6 ± 2.3	14.2 ± 1.6	15.8 ± 3.1	13.7 ± 0.9
CRE (mg/dL)	0.37 ± 0.01	0.40 ± 0.05	0.36 ± 0.03	0.36 ± 0.03	0.42 ± 0.08	0.39 ± 0.02	0.37 ± 0.01	0.37 ± 0.04
IP (mg/dL)	8.95 ± 0.29	8.96 ± 0.31	8.79 ± 0.21	8.52 ± 0.22	7.88 ± 0.19	7.88 ± 0.41	7.52 ± 0.48	7.49 ± 0.51
Ca^2+^ (mmol/L)	9.80 ± 0.13	9.76 ± 0.11	9.87 ± 0.20	9.65 ± 0.30	9.85 ± 0.15	9.86 ± 0.12	9.90 ± 0.12	9.91 ± 0.07
Na^+^ (mmol/L)	141.8 ± 2.8	143.0 ± 0.8	141.9 ± 0.9	141.5 ± 0.6	140.2 ± 0.8	140.5 ± 1.3	141.5 ± 0.4	140.7 ± 0.5
K^+^ (mmol/L)	5.05 ± 0.57	4.91 ± 0.37	4.99 ± 0.17	4.87 ± 0.56	4.84 ± 0.34	4.84 ± 0.13	4.62 ± 0.08	4.41 ± 0.14
Cl^−^ (mmol/L)	98.9 ± 2.8	101.0 ± 2.0	99.8 ± 0.9	99.9 ± 1.0	103.5 ± 1.1	103.7 ± 1.5	103.9 ± 1.3	102.6 ± 1.2

G1: negative control; G2: 500 mg/kg OE treated group; G3: 1000 mg/kg OE treated group; G4: 2000 mg/kg OE treated group

AST: aspartate aminotransferase, ALT: alanine aminotransferase, ALP: alkaline phosphatase, CPK: creatinine phosphokinase, TBIL: Total bilirubin, GLU: glucose, TCHO: total cholesterol, TG: triglyceride, TP: total protein, ALB: albumin, A/G: Albumin/Globulin, BUN: blood urea nitrogen, CRE: creatinine, IP: inorganic phosphorus

Data are expressed as mean ± SD. **Significant difference at *P* < 0.01 levels compared with the vehicle control. Bonferroni’s post hoc test was used following a one-way ANOVA vs. G1, and the homogeneity of variance was tested using Levene’s test

### Genotoxicity test

#### Bacterial reverse mutation test (Ames test)

No precipitation was observed in any of the concentration groups when mixing and colony counting were performed with the preparation and top agar. There was no colonization due to microbial contamination on the plates used to confirm the highest OE concentration and the sterility of the S9 mix. No decrease in the colony number and cytotoxicity were observed in any of the strains with the increasing OE concentrations. In measuring the cell numbers of the 5 strains used in the test, the number of viable treated cells per plate was 0.91–1.13 × 10^9^ (TA strain) and 1.17 × 10^9^ (*E. coli*) CFU/mL. The average number of colonies in the tested, substance-treated group did not show any increase regardless of whether the metabolic active system was applied in all of the test strains (Table 5). Therefore, the OE did not induce a mutation in the test strain.

**Table 5:** Bacterial reverse mutation assay

Test Strain	Chemical Treated	Dose (μg/plate)	Colonies/plate [factor]^a^	
			With S9 mix	Without S9 mix
TA100	Test article	0	114 ± 13	109 ± 3
		5	130 ± 7 [1.1]	115 ± 11 [1.1]
		15	98 ± 9 [0.9]	104 ± 0 [1.0]
		50	117 ± 13 [1.0]	113 ± 14 [1.0]
		150	126 ± 9 [1.1]	115 ± 10 [1.1]
		500	116 ± 12 [1.0]	116 ± 3 [1.1]
		1500	121 ± 0 [1.1]	121 ± 7 [1.1]
		3000	133 ± 4 [1.2]	120 ± 11[1.1]
		5000	134 ± 3 [1.2]	104 ± 6 [1.0]
TA1535	Test article	0	12 ± 1	12 ± 2
		5	11 ± 1 [0.9]	11 ± 1 [0.9]
		15	10 ± 1 [0.8]	12 ± 4 [1.0]
		50	12 ± 1 [1.0]	13 ± 3 [1.1]
		150	9 ± 1 [0.7]	10 ± 1 [0.8]
		500	12 ± 3 [1.0]	14 ± 4 [1.2]
		1500	11 ± 1 [0.9]	14 ± 4 [1.2]
		3000	9 ± 0 [0.8]	10 ± 1 [0.9]
		5000	12 ± 3 [1.0]	11 ± 2 [0.9]
TA98	Test article	0	26 ± 4	24 ± 4
		5	27 ± 4 [1.0]	20 ± 1 [0.8]
		15	23 ± 2 [0.9]	21 ± 1 [0.9]
		50	25 ± 2 [1.0]	26 ± 6 [1.1]
		150	27 ± 4 [1.0]	25 ± 1 [1.0]
		500	30 ± 2 [1.2]	24 ± 3 [1.0]
		1500	27 ± 1 [1.1]	23 ± 1 [1.0]
		3000	29 ± 1 [1.1]	23 ± 3 [1.0]
		5000	31 ± 4 [1.2]	27 ± 4 [1.1]
TA1537	Test article	0	11 ± 1	9 ± 1
		5	10 ± 2 [0.9]	8 ± 0 [0.9]
		15	10 ± 1 [0.9]	8 ± 3 [0.9]
		50	10 ± 2 [0.9]	9 ± 4 [1.0]
		150	11 ± 4 [1.0]	7 ± 2 [0.8]
		500	14 ± 4 [1.3]	8 ± 2 [0.9]
		1500	12 ± 2 [1.1]	7 ± 1 [0.8]
		3000	9 ± 0 [0.9]	7 ± 1 [0.8]
		5000	9 ± 0 [0.9]	7 ± 1 [0.8]
*E. coli* WP2 uvrA	Test article	0	29 ± 0	22 ± 4
		5	23 ± 1 [0.8]	27 ± 1 [1.2]
		15	24 ± 4 [0.8]	23 ± 4 [1.1]
		50	29 ± 4 [1.0]	23 ± 3 [1.1]
		150	26 ± 1 [0.9]	21 ± 3 [1.0]
		500	25 ± 1 [0.9]	26 ± 2 [1.2]
		1500	25 ± 6 [0.9]	24 ± 4 [1.1]
		3000	26 ± 0 [0.9]	21 ± 4 [1.0]
		5000	23 ± 1 [0.8]	26 ± 8 [1.2]
Positive controls		(μg/plate)		
TA100	2-AA	1.0	1588 ± 14 [13.7]	
TA1535	2-AA	2.0	183 ± 2 [15.2]	
TA98	B[a]P	1.0	159 ± 6 [6.2]	
TA1537	2-AA	1.0	239 ± 1 [22.8]	
WP2 uvrA	2-AA	6.0	143 ± 11 [4.9]	
TA100	SA	0.5		408 ± 5 [3.7]
TA1535	SA	0.5		286 ± 6 [24.8]
TA98	2-NF	2.0		208 ± 11[8.7]
TA1537	ICR-191	0.5		59 ± 1 [6.9]
WP2 uvrA	4NQO	0.5		167 ± 9 [7.7]

Test article: Extract of 70% extract of palm cactus stem

^a^Two plate/dose were used. No. of colonies of treated plate/No. of colonies of the negative control plate

Abbreviations: 2-AA: 2-aminoanthracene, SA: sodium azide, B[a]P: benzo[a]pyrene, ICR-191: acridine mutagen ICR 191, 4NQQ: 4-nitroqinoline N-oxide, 2-NF: 2-Nitrofluorene

#### Chromosome aberration test using CHL cells

When treated with the OE, turbidity was observed in concentration groups of 1500 μg/mL or greater, and turbidity and precipitation were observed in the 5000 μg/mL concentration group. The frequency of structural abnormalities was less than 2.00% in the negative control group and all the OE treatment groups when the OE was applied for 6 h under the metabolic activation system. No increase in the number of colonies was observed in all the concentration groups treated with the test substance. At that time, the frequency of the mid-term phase with structural abnormalities in the positive control group was 30.00%. The frequency of structural abnormalities was less than 2.00% in the negative control group and all the OE treatment groups when the OE was applied for 6 h without the metabolic activation system. No increase in the number of colonies was observed in all the concentration groups treated with the test substance. At that time, the frequency of the middle phase in the positive control group was 18.00%. The frequency of structural abnormalities was less than 1.00% in the negative control group and all the OE treatment groups when the OE was applied for 24 h without the metabolic activation system, and no increase in the number of colonies was observed in all the concentration groups treated with the test substance. At that time, the frequency of the middle phase for the positive control group was 21.00% (Table 6). The 70% alcohol extract from the OFIS stem does not induce a chromosomal abnormality in the CHL cells.

**Table 6:** In vitro chromosome aberration test in chinese hamster lung cells with OE extract from cactus stem

Treatment Schedule^a^	Dose (μg/mL)		Ratio of Aberrant Metaphase^b^	Cell Counts^c^	Mean		RICC(%)^d^
6–18 (+S)	0		0	4792	4689	4741	100
	500		0	5024	4931	4978	108
	1500	P	0	4996	4774	4885	105
	3000	P	2	4405	4247	4326	86
	3500	P	2	4158	4107	4133	80
	4000	P	1	3528	3491	3510	59
	4500	TTP	1	3054	2971	3013	42
	5000	TTP	2	2796	2756	2776	34
	B[a]P 20		30	3066	3116	3091	45
6–18 (-S)	0		0	5899	5757	5828	100
	500		0	5525	5389	5457	91
	1500	P	1	5001	4962	4982	79
	1800	P	2	4243	4539	4391	65
	2000	P	0	3508	3445	3477	42
	3000	P	2	2942	2734	2838	26
	4NQO 0.4		18	3226	3148	3187	35
24–0 (-S)	0		0	5600	5444	5522	100
	500		1	5400	5479	5440	98
	1500	P	1	4971	4971	4971	85
	3000	P	1	4153	4072	4113	63
	4000	P	0	3863	3842	3853	56
	5000	TTP	0	3652	3670	3661	51
	4NQO 0.4		21	2853	2994	2924	31
Initial cell count		Cell counts^c^				Mean	
		1754	1673	1872	1750	1762	

^a^Treatment time – Recovery time in the presence (+S) and absence (-S) of metabolic activation system

^b^Ratio of metaphase with chromosome aberrations. One culture/ dose was used

Gaps excludes, 100 metaphases/ culture were examined

^c^After harvesting mitotic cells, each culture was trypsinized and suspended with 0.5 mL of 0.1% trypsin and 5 mL of culture medium

The cell suspensions of 0.4 mL per culture were diluted 50 times with 19.6 mL of Isoton sol. The cells in 0.5 mL Isoton sol. were counted twice/ culture using Coulter Counter model Z2

Actual number of cells per flask = Mean Count × 550

^d^Relative Increase in cell Count = ((Cell count of treated flask – Initial cell count) / (Cell count of the negative control flask – initial cell count)) × 100 (%)

T: Turbidity at the end of the treatment

P: Precipitation at the end of the treatment

B[a]: benzo[a] pyrene, 4NQO: 4-Nitroquinoline-1-oxide

### Micronucleus test using mouse bone marrow cells

No change in body weight or gross abnormality was observed in the OE administration group. The micronucleated polychromatic erythrocytes (MNPCE) frequencies observed in the 2000 PCEs per subject were 1.00, 2.25, 1.50, 1.50, and 1.00 for the negative control at OE 500, 1000, 2000, and 5000 mg/kg/day, respectively. There was no statistically significant increase in the tested, substance-treated group compared with the negative control group; in addition, there was no dose-related correlation, and the result was within the HCD range (Table 7). At that time, the MNPCE frequency of the positive control group was 78.25. The PCE: RBC ratios, which are an indicator of cytotoxicity, were 0.52, 0.58, 0.62, 0.55, and 0.56 for the negative control group and the 500, 1000, 2000, and 5000 mg/kg groups, respectively, but none was significantly decreased. The PCE: RBC ratio of the positive control group was 0.52, and there was no statistically significant change. OE did not induce micronuclei in the bone marrow cells of male ICR mice under these test conditions.

**Table 7:** Observations of micronucleus and PCE: RBC ratio – summary

Dose (mg/kg/day)	Animals per Dose	MNPCE/2000 PCE	PCE:RBC Ratio	(% Control)
0	4	1.00 ± 0.82	0.52 ± 0.05	100
500	4	2.25 ± 1.26	0.58 ± 0.07	110
1000	4	1.50 ± 1.29	0.62 ± 0.04	119
2000	4	1.50 ± 1.00	0.55 ± 0.08	104
5000	4	1.00 ± 0.82	0.56 ± 0.08	106
CPA 70	4	78.25 ± 4.86*	0.52 ± 0.06	99

OE: *Opuntia ficus-indica var. saboten* extract, MNPCE: Micronucleated polychromatic erythrocyte, PCE: Polychromatic erythrocyte, RBC: Red blood cells (polychromatic erythrocyte+normochromatic erythrocyte), CPA: Cyclophosphamide monohydrate (positive control article)

The data are reported as the mean ± SD. The non-parametric Kruskal-Wallis test was performed on the frequency of micronucleus using SPSS Statistics 22 for Medical Science in the micronucleus test, and the negative and positive controls data were analyzed using the Mann-Whitney U test. *Significantly different from the negative control group at *P* < 0.05

## Discussion

The results of the present animal toxicity study revealed that a one-week oral administration of OE at levels up to 2000 mg/kg in male and female Sprague Dawley rats was not associated with toxic effects as evaluated by the general symptoms of the animals, including their growth, feed and water consumption, functional observations, hematology, serum biochemical profiles, urinalysis, organ weights, ophthalmological examination, and pathology findings. However, few results differed statistically from the vehicle-treated group, but all the data were within the reference values for Sprague Dawley rats.

All the hematological profiles were normal among all the treated groups in comparison with the vehicle-treated group, except the RDW. This parameter was significantly higher only in females treated with 2000 mg/kg/day in comparison with vehicle-treated female rats, but it was estimated to be within the range of historical reference data. The RDW represents the variability in the size of circulating red blood cells (anisocytosis). This measurement is an important tool in the complete blood count for diagnosing certain anemias (e.g., sickle cell anemia, hemolysis or hemolytic anemias), especially those that are microcytic and caused by iron/folic acid or vitamin B12 deficiencies. Another cause of RDW elevation may result from the premature release of immature RBC into the bloodstream due to severe blood loss [17]. However, all of the elevated RDW cases were accompanied by the alteration of other blood cells, especially RBC and hemoglobin, which was not found in the treated groups. Therefore, an elevated RDW might be associated with individual biological discrimination rather than the adverse effects of OE.

The serum BUN values were significantly higher in males but not in female rats when compared with vehicle-treated male and female rats, respectively. The serum BUN and CRE were elevated following kidney injury or abnormal protein catabolism [18]. However, there was no difference in the CRE levels in male and female rats. The serum TP and albumin levels were also normal, indicating the absence of abnormal protein catabolism. In addition, if the BUN elevation occurred due to the toxic effect of OE, higher doses of OE should cause these effects. The peak BUN in male rats was 14.9 ± 1.5 mg/dL and was found after the medium dose (1000 mg/kg) of OE, while 13.2 ± 0.8 mg/dL and 13.0 ± 1.4 mg/dL (lowest level of BUN) were found for the 500 and 2000 mg/kg doses, respectively. Therefore, dose-response correlations were not observed. The autopsy findings showed no change in long-term weight due to the OE, and no gross abnormal kidney findings were observed. Blood biochemical tests showed that the TBIL and ALT levels were significantly higher in females treated with 2000 mg/kg of OE (Tables 2, 3) but not in male rats. These alterations were also not associated with the dosage. The elevated TBIL and ALT apparently indicated liver toxicity. However, if hepatotoxicity occurs, the feed and water consumption, body weight gain, and liver weight are also altered following treatment administration [19, 20]. Interestingly, these parameters were not altered. In addition, there were no differences between the urine BIL levels in the treated groups, in both female and male rats, and in the vehicle-treated groups. Furthermore, the other cytosolic enzymes (i.e., AST and ALP) were similar for the treated and vehicle-control groups, both for male and female rats. OE apparently exerts hepatoprotective effects and protects the organophosphorus-induced alteration in the liver weight and morphology, serum AST, ALT, albumin, and cholesterol levels [21]. Nevertheless, the TG levels were significantly lower in female rats treated with 1000 mg/kg but not the 2000 mg/kg group. Dose-response correlations were not observed. In addition, another lipid profile (i.e., TCHO) was not statistically changed between the untreated and vehicle-treated groups for both male and female rats. Therefore, the close observation of clinical, behavioral, hematological, and serum biochemical profiles suggested that any observed variations were most likely due to incidental biological variations, and none were associated with harmful effects from OE in rats.

The molecular toxicity of OE was tested by bacterial reverse mutation test (Ames assay), in vitro chromosomal aberration test in CHL cells, and in vivo mammalian micronucleus test. The results showed that the OE did not reveal any genotoxic action. For the Ames test, the histidine operon-containing strains, *S. typhimurium* TA100, TA1535, TA98, and TA1537 [13] and tryptophan strain *E. coli* WP2 urvA [14] were used to detect the base pair substitution mutations or frameshift mutations and showed that OE had no mutagenic effects. In addition, to detect cellular DNA damage at the chromosomal level, the in vitro chromosomal aberration test and in vivo micronucleus test were performed. The results of these tests indicated that OE had no mutagenic or clastogenic effects. The oral administration of the OE also caused no significant increase in the number of MNPCE or in the mean ratio of polychromatic to total erythrocytes, which further ensured that the molecular effects were nontoxic.

## Conclusions

In summary, the results of the present in vivo toxicity study suggest that the oral administration of OE at levels up to 2000 mg/kg/day does not cause adverse effects in male and female rats. The results of the mutagenicity studies suggest that OE is not genotoxic over the dose ranges tested in this experiment. Therefore, in the present investigations, the in vivo and in vitro tests indicate that OE could be considered as a reliable and safe herbal medicine or functional food due to the absence of any toxicity-related findings under the conditions of the study.

## Abbreviations

2-AA: 2-Aminoanthracene
2-NF: 2-Nitrofluorene
4-NQO: 4-Nitroquinoline-1-oxide
ALT: Alanine aminotransferase
AST: Aspartate aminotransferase
BUN: Blood urea nitrogen
CFU: Colony-forming unit
CHL: Chinese hamster lung
CPA: Cyclophosphamide monohydrate
CRE: Creatinine
HCD: Historical control data
MEM: Minimal essential medium
MNPCE: Micronucleated polychromatic erythrocytes
OE: *Opuntia ficus-indica* var. *saboten* extract
OFIS: *Opuntia ficus-indica* var. *saboten*
PCE: Polychromatic erythrocyte
RBC: Red blood cell
RDW: Red blood cell distribution width
TBIL: Total bilirubin
TCHO: Total cholesterol
TG: Triglyceride
TP: Total protein
